# 1-[5-(4-Chloro­phen­yl)-3-(4-hy­droxy­phen­yl)-4,5-dihydro-1*H*-pyrazol-1-yl]­ethanone

**DOI:** 10.1107/S1600536812006885

**Published:** 2012-02-24

**Authors:** Hoong-Kun Fun, Ching Kheng Quah, S. Priya, B. Narayana, B. K. Sarojini

**Affiliations:** a X-ray Crystallography Unit, School of Physics, Universiti Sains Malaysia, 11800 USM, Penang, Malaysia; b Department of Studies in Chemistry, Mangalore University, Mangalagangotri 574 199, India; c Department of Chemistry, P. A. College of Engineering, Nadupadavu, Mangalore 574 153, India

## Abstract

In the title compound, C_17_H_15_ClN_2_O_2_, the benzene rings form dihedral angles of 89.56 (5) and 5.87 (5)° with the mean plane of the pyrazoline ring (r.m.s. deviation = 0.084 Å). The dihedral angle between the two benzene rings is 87.57 (5)°. In the crystal, mol­ecules are linked by O—H⋯O and C—H⋯O hydrogen bonds into a helical chain along the *c* axis. Between the chains weak C—H⋯N and C—H⋯O inter­actions are present. The crystal studied was an inversion twin with a domain ratio of 0.72 (4):0.28 (4).

## Related literature

For general background to and the biological activities of pyrazolines, see: Samshuddin *et al.* (2011 ▶); Sarojini *et al.* (2010 ▶). For standard bond-length data, see: Allen *et al.* (1987 ▶). For the stability of the temperature controller used for the data collection, see: Cosier & Glazer (1986 ▶). For a related structure, see: Fun *et al.* (2010 ▶).
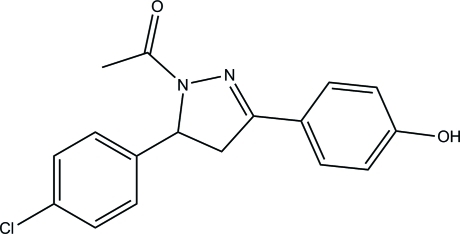


## Experimental

### 

#### Crystal data

C_17_H_15_ClN_2_O_2_
*M* *_r_* = 314.76
Orthorhombic, 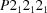

*a* = 5.0213 (2) Å
*b* = 15.6834 (5) Å
*c* = 18.6368 (6) Å
*V* = 1467.67 (9) Å^3^
*Z* = 4
Mo *K*α radiation
μ = 0.27 mm^−1^
*T* = 100 K
0.43 × 0.36 × 0.22 mm

#### Data collection

Bruker SMART APEXII CCD area-detector diffractometer
Absorption correction: multi-scan (*SADABS*; Bruker, 2009 ▶) *T*_min_ = 0.892, *T*_max_ = 0.944
21739 measured reflections
5194 independent reflections
4925 reflections with *I* > 2σ(*I*)
*R*_int_ = 0.019

#### Refinement

*R*[*F* ^2^ > 2σ(*F* ^2^)] = 0.030
*wR*(*F* ^2^) = 0.082
*S* = 1.05
5194 reflections
205 parameters
H atoms treated by a mixture of independent and constrained refinement
Δρ_max_ = 0.33 e Å^−3^
Δρ_min_ = −0.26 e Å^−3^
Absolute structure: Flack (1983 ▶), 2089 Friedel pairs
Flack parameter: 0.28 (4)

### 

Data collection: *APEX2* (Bruker, 2009 ▶); cell refinement: *SAINT* (Bruker, 2009 ▶); data reduction: *SAINT*; program(s) used to solve structure: *SHELXTL* (Sheldrick, 2008 ▶); program(s) used to refine structure: *SHELXTL*; molecular graphics: *SHELXTL*; software used to prepare material for publication: *SHELXTL* and *PLATON* (Spek, 2009 ▶).

## Supplementary Material

Crystal structure: contains datablock(s) global, I. DOI: 10.1107/S1600536812006885/is5074sup1.cif

Structure factors: contains datablock(s) I. DOI: 10.1107/S1600536812006885/is5074Isup2.hkl

Supplementary material file. DOI: 10.1107/S1600536812006885/is5074Isup3.cml

Additional supplementary materials:  crystallographic information; 3D view; checkCIF report

## Figures and Tables

**Table 1:** Hydrogen-bond geometry (Å, °)

*D*—H⋯*A*	*D*—H	H⋯*A*	*D*⋯*A*	*D*—H⋯*A*
O1—H1*O*1⋯O2^i^	0.88 (2)	1.82 (2)	2.6971 (12)	171 (2)
C8—H8*A*⋯N2^ii^	0.99	2.56	3.5038 (14)	160
C8—H8*B*⋯O1^iii^	0.99	2.52	3.4887 (12)	166
C12—H12*A*⋯O2^i^	0.95	2.51	3.1982 (12)	130

Symmetry codes: (i) 
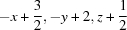
; (ii) 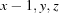; (iii) 
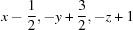
.

**Fig. 1.**
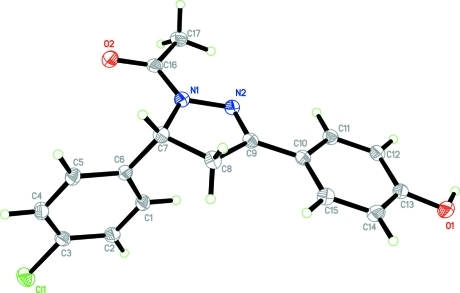
The molecular structure of the title compound, showing 50% probability displacement ellipsoids for non-H atoms.

**Fig. 2.**
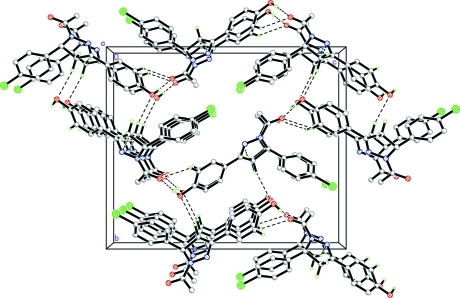
The packing diagram of the title compound, viewed along the a axis. H atoms not involved in hydrogen bonds (dashed lines) have been omitted for clarity.

## supplementary crystallographic information

### Comment

Pyrazolines have been reported to exhibit a broad spectrum of biological activities including antibacterial, antifungal, antioxidant and analgesic properties (Samshuddin *et al.*, 2011; Sarojini *et al.*, 2010). In continuation of our work on synthesis of pyrazoline derivatives (Fun *et al.*, 2010), the title compound (I) is prepared and its crystal structure is reported.

In the title molecule (Fig. 1), the two benzene rings (C1–C6 and C10–C15) form dihedral angles of 89.56 (5) and 5.87 (5)°, respectively, with the 4,5-dihydro-1*H*-pyrazole ring (N1/N2/C7-C9). The benzene rings form a dihedral angle of 87.57 (5)°. Bond lengths (Allen *et al.*, 1987) and angles are within normal ranges and are comparable with a related structure (Fun *et al.*, 2010). The crystal studied was an inversion twin with a domain ratio of 0.28 (4):0.72 (4). In the crystal structure (Fig. 2) molecules are linked *via* intermolecular O1—H1O1···O2 and C12—H12A···O2 hydrogen bonds (Table 1) into a helical chain along the *c* axis. Weak C8—H8A···N2 and C8—H8B···O1 are also observed between the chains.

### Experimental

A mixture of (2*E*)-3-(4-chlorophenyl)-1-(4-hydroxyphenyl)prop-2-en-1-one (2.58 g, 0.01 mol) and hydrazine hydrate (0.5 ml, 0.01 mol) in 25 ml acetic acid was refluxed for 6 h. The reaction mixture was cooled and poured into 50 ml ice-cold water. The precipitate was collected by filtration and purified by recrystallization from ethanol. The single crystals were grown from DMF by slow evaporation method and yield of the compound was 80% (*m.p.* : 530 K).

### Refinement

Atom H1O1 was located in a difference Fourier map and refined freely [refined distance O1—H1O1 = 0.88 (2) Å]. The remaining H atoms were positioned geometrically and refined using a riding model, with C—H = 0.95 or 1.00 Å and *U*_iso_(H) = 1.2 or 1.5 *U*_eq_(C). A rotating group model was applied to the methyl group. The crystal studied was an inversion twin with a 0.28 (4):0.72 (4) domain ratio.

### Crystal data

**Table:**

C_17_H_15_ClN_2_O_2_	*F*(000) = 656
*M**_r_* = 314.76	*D*_x_ = 1.424 Mg m^−^^3^
Orthorhombic, *P*2_1_2_1_2_1_	Mo *K*α radiation, λ = 0.71073 Å
Hall symbol: P 2ac 2ab	Cell parameters from 9904 reflections
*a* = 5.0213 (2) Å	θ = 3.4–32.7°
*b* = 15.6834 (5) Å	µ = 0.27 mm^−^^1^
*c* = 18.6368 (6) Å	*T* = 100 K
*V* = 1467.67 (9) Å^3^	Block, colourless
*Z* = 4	0.43 × 0.36 × 0.22 mm

### Data collection

**Table:**

Bruker SMART APEXII CCD area-detector diffractometer	5194 independent reflections
Radiation source: fine-focus sealed tube	4925 reflections with *I* > 2σ(*I*)
Graphite monochromator	*R*_int_ = 0.019
φ and ω scans	θ_max_ = 32.7°, θ_min_ = 1.7°
Absorption correction: multi-scan (*SADABS*; Bruker, 2009)	*h* = −7→7
*T*_min_ = 0.892, *T*_max_ = 0.944	*k* = −23→23
21739 measured reflections	*l* = −28→28

### Refinement

**Table:**

Refinement on *F*^2^	Secondary atom site location: difference Fourier map
Least-squares matrix: full	Hydrogen site location: inferred from neighbouring sites
*R*[*F*^2^ > 2σ(*F*^2^)] = 0.030	H atoms treated by a mixture of independent and constrained refinement
*wR*(*F*^2^) = 0.082	*w* = 1/[σ^2^(*F*_o_^2^) + (0.0486*P*)^2^ + 0.1907*P*] where *P* = (*F*_o_^2^ + 2*F*_c_^2^)/3
*S* = 1.05	(Δ/σ)_max_ = 0.001
5194 reflections	Δρ_max_ = 0.33 e Å^−^^3^
205 parameters	Δρ_min_ = −0.26 e Å^−^^3^
0 restraints	Absolute structure: Flack (1983), 2089 Friedel pairs
Primary atom site location: structure-invariant direct methods	Flack parameter: 0.28 (4)

### Special details

**Table:**

Experimental. The crystal was placed in the cold stream of an Oxford Cryosystems Cobra open-flow nitrogen cryostat (Cosier & Glazer, 1986) operating at 100.0 (1) K.
Geometry. All esds (except the esd in the dihedral angle between two l.s. planes) are estimated using the full covariance matrix. The cell esds are taken into account individually in the estimation of esds in distances, angles and torsion angles; correlations between esds in cell parameters are only used when they are defined by crystal symmetry. An approximate (isotropic) treatment of cell esds is used for estimating esds involving l.s. planes.
Refinement. Refinement of F^2^ against ALL reflections. The weighted R-factor wR and goodness of fit S are based on F^2^, conventional R-factors R are based on F, with F set to zero for negative F^2^. The threshold expression of F^2^ > 2sigma(F^2^) is used only for calculating R-factors(gt) etc. and is not relevant to the choice of reflections for refinement. R-factors based on F^2^ are statistically about twice as large as those based on F, and R- factors based on ALL data will be even larger.

### Fractional atomic coordinates and isotropic or equivalent isotropic displacement parameters (Å^2^)

**Table:**

	*x*	*y*	*z*	*U*_iso_*/*U*_eq_	
Cl1	0.69906 (7)	0.812228 (18)	0.062645 (13)	0.02929 (7)	
O1	0.64339 (19)	0.76579 (5)	0.68963 (4)	0.02423 (17)	
O2	0.49802 (19)	1.14641 (5)	0.26805 (4)	0.02311 (16)	
N1	0.48592 (19)	1.05034 (5)	0.35670 (4)	0.01842 (16)	
N2	0.57563 (19)	1.01940 (5)	0.42259 (4)	0.01797 (16)	
C1	0.5989 (2)	0.89416 (6)	0.26375 (5)	0.01860 (17)	
H1A	0.6798	0.8854	0.3092	0.022*	
C2	0.6960 (2)	0.85102 (6)	0.20404 (5)	0.01995 (18)	
H2A	0.8422	0.8129	0.2084	0.024*	
C3	0.5754 (2)	0.86468 (6)	0.13803 (5)	0.01985 (19)	
C4	0.3600 (2)	0.91916 (7)	0.13069 (5)	0.0213 (2)	
H4A	0.2775	0.9270	0.0853	0.026*	
C5	0.2664 (2)	0.96220 (6)	0.19103 (5)	0.01969 (18)	
H5A	0.1202	1.0002	0.1865	0.024*	
C6	0.3843 (2)	0.95017 (6)	0.25775 (5)	0.01623 (16)	
C7	0.2786 (2)	0.99601 (6)	0.32343 (5)	0.01790 (17)	
H7A	0.1201	1.0313	0.3103	0.021*	
C8	0.2094 (2)	0.93621 (6)	0.38672 (5)	0.01917 (17)	
H8A	0.0320	0.9497	0.4069	0.023*	
H8B	0.2129	0.8757	0.3717	0.023*	
C9	0.4279 (2)	0.95519 (6)	0.43990 (5)	0.01668 (16)	
C10	0.4788 (2)	0.90758 (6)	0.50616 (5)	0.01641 (16)	
C11	0.6865 (2)	0.93201 (6)	0.55202 (5)	0.01882 (17)	
H11A	0.7907	0.9807	0.5405	0.023*	
C12	0.7424 (2)	0.88630 (6)	0.61378 (5)	0.01932 (18)	
H12A	0.8841	0.9036	0.6443	0.023*	
C13	0.5895 (2)	0.81446 (6)	0.63127 (5)	0.01882 (17)	
C14	0.3774 (2)	0.79110 (7)	0.58721 (6)	0.02158 (19)	
H14A	0.2688	0.7438	0.5997	0.026*	
C15	0.3245 (2)	0.83700 (6)	0.52498 (5)	0.01973 (18)	
H15A	0.1813	0.8201	0.4949	0.024*	
C16	0.5891 (2)	1.12119 (6)	0.32603 (5)	0.01928 (18)	
C17	0.8134 (3)	1.16620 (7)	0.36378 (6)	0.0243 (2)	
H17A	0.9646	1.1723	0.3309	0.037*	
H17B	0.8681	1.1329	0.4058	0.037*	
H17C	0.7536	1.2228	0.3792	0.037*	
H1O1	0.774 (5)	0.7911 (13)	0.7133 (11)	0.056 (6)*	

### Atomic displacement parameters (Å^2^)

**Table:**

	*U*^11^	*U*^22^	*U*^33^	*U*^12^	*U*^13^	*U*^23^
Cl1	0.03723 (16)	0.03089 (13)	0.01976 (10)	−0.00079 (12)	0.00636 (10)	−0.00525 (9)
O1	0.0309 (4)	0.0200 (3)	0.0218 (3)	−0.0001 (3)	−0.0040 (3)	0.0028 (3)
O2	0.0285 (4)	0.0202 (3)	0.0205 (3)	0.0030 (3)	0.0016 (3)	0.0018 (3)
N1	0.0205 (4)	0.0169 (3)	0.0178 (3)	0.0003 (3)	−0.0021 (3)	−0.0003 (3)
N2	0.0198 (4)	0.0170 (3)	0.0171 (3)	0.0013 (3)	−0.0008 (3)	−0.0004 (3)
C1	0.0185 (4)	0.0194 (4)	0.0179 (4)	0.0021 (4)	−0.0021 (3)	0.0006 (3)
C2	0.0202 (5)	0.0187 (4)	0.0210 (4)	0.0020 (4)	0.0003 (4)	−0.0008 (3)
C3	0.0241 (5)	0.0180 (4)	0.0174 (4)	−0.0041 (4)	0.0032 (4)	−0.0007 (3)
C4	0.0239 (5)	0.0229 (4)	0.0170 (4)	−0.0021 (4)	−0.0023 (4)	0.0017 (3)
C5	0.0192 (4)	0.0204 (4)	0.0195 (4)	0.0018 (4)	−0.0025 (4)	0.0022 (3)
C6	0.0153 (4)	0.0162 (4)	0.0173 (3)	−0.0005 (3)	−0.0008 (3)	0.0007 (3)
C7	0.0159 (4)	0.0186 (4)	0.0192 (3)	0.0019 (4)	−0.0012 (3)	−0.0004 (3)
C8	0.0154 (4)	0.0236 (4)	0.0185 (3)	−0.0011 (4)	0.0005 (3)	−0.0010 (3)
C9	0.0149 (4)	0.0181 (4)	0.0170 (3)	0.0019 (3)	0.0004 (3)	−0.0025 (3)
C10	0.0151 (4)	0.0173 (4)	0.0168 (3)	0.0013 (3)	0.0012 (3)	−0.0014 (3)
C11	0.0193 (4)	0.0187 (4)	0.0184 (4)	−0.0017 (4)	0.0003 (3)	−0.0012 (3)
C12	0.0200 (5)	0.0201 (4)	0.0178 (3)	−0.0006 (4)	−0.0001 (3)	−0.0020 (3)
C13	0.0217 (4)	0.0167 (4)	0.0181 (3)	0.0032 (4)	0.0016 (3)	−0.0015 (3)
C14	0.0222 (5)	0.0185 (4)	0.0240 (4)	−0.0027 (4)	0.0004 (4)	0.0014 (3)
C15	0.0174 (4)	0.0202 (4)	0.0216 (4)	−0.0002 (4)	0.0002 (4)	−0.0004 (3)
C16	0.0232 (5)	0.0154 (4)	0.0192 (4)	0.0024 (4)	0.0044 (4)	−0.0020 (3)
C17	0.0306 (5)	0.0177 (4)	0.0247 (4)	−0.0042 (4)	0.0007 (4)	−0.0025 (3)

### Geometric parameters (Å, º)

**Table:**

Cl1—C3	1.7425 (10)	C7—H7A	1.0000
O1—C13	1.3560 (12)	C8—C9	1.5080 (14)
O1—H1O1	0.88 (2)	C8—H8A	0.9900
O2—C16	1.2382 (13)	C8—H8B	0.9900
N1—C16	1.3527 (13)	C9—C10	1.4656 (13)
N1—N2	1.3951 (11)	C10—C15	1.3961 (14)
N1—C7	1.4812 (14)	C10—C11	1.4018 (14)
N2—C9	1.2917 (13)	C11—C12	1.3847 (13)
C1—C2	1.3906 (14)	C11—H11A	0.9500
C1—C6	1.3947 (14)	C12—C13	1.4019 (15)
C1—H1A	0.9500	C12—H12A	0.9500
C2—C3	1.3878 (14)	C13—C14	1.3937 (15)
C2—H2A	0.9500	C14—C15	1.3906 (14)
C3—C4	1.3853 (17)	C14—H14A	0.9500
C4—C5	1.3932 (14)	C15—H15A	0.9500
C4—H4A	0.9500	C16—C17	1.5039 (17)
C5—C6	1.3901 (13)	C17—H17A	0.9800
C5—H5A	0.9500	C17—H17B	0.9800
C6—C7	1.5156 (13)	C17—H17C	0.9800
C7—C8	1.5464 (14)		
			
C13—O1—H1O1	107.2 (13)	C7—C8—H8B	111.2
C16—N1—N2	122.28 (9)	H8A—C8—H8B	109.2
C16—N1—C7	124.39 (8)	N2—C9—C10	120.49 (9)
N2—N1—C7	113.28 (8)	N2—C9—C8	114.05 (9)
C9—N2—N1	107.79 (8)	C10—C9—C8	125.46 (9)
C2—C1—C6	120.88 (9)	C15—C10—C11	118.46 (9)
C2—C1—H1A	119.6	C15—C10—C9	121.25 (9)
C6—C1—H1A	119.6	C11—C10—C9	120.29 (9)
C3—C2—C1	118.76 (10)	C12—C11—C10	121.07 (10)
C3—C2—H2A	120.6	C12—C11—H11A	119.5
C1—C2—H2A	120.6	C10—C11—H11A	119.5
C4—C3—C2	121.58 (9)	C11—C12—C13	119.89 (10)
C4—C3—Cl1	119.33 (8)	C11—C12—H12A	120.1
C2—C3—Cl1	119.10 (9)	C13—C12—H12A	120.1
C3—C4—C5	118.84 (9)	O1—C13—C14	118.50 (9)
C3—C4—H4A	120.6	O1—C13—C12	121.97 (10)
C5—C4—H4A	120.6	C14—C13—C12	119.53 (9)
C6—C5—C4	120.85 (10)	C15—C14—C13	120.08 (10)
C6—C5—H5A	119.6	C15—C14—H14A	120.0
C4—C5—H5A	119.6	C13—C14—H14A	120.0
C5—C6—C1	119.09 (9)	C14—C15—C10	120.93 (10)
C5—C6—C7	120.59 (9)	C14—C15—H15A	119.5
C1—C6—C7	120.31 (8)	C10—C15—H15A	119.5
N1—C7—C6	111.39 (9)	O2—C16—N1	119.33 (10)
N1—C7—C8	100.81 (7)	O2—C16—C17	122.34 (10)
C6—C7—C8	114.01 (8)	N1—C16—C17	118.33 (9)
N1—C7—H7A	110.1	C16—C17—H17A	109.5
C6—C7—H7A	110.1	C16—C17—H17B	109.5
C8—C7—H7A	110.1	H17A—C17—H17B	109.5
C9—C8—C7	102.60 (8)	C16—C17—H17C	109.5
C9—C8—H8A	111.2	H17A—C17—H17C	109.5
C7—C8—H8A	111.2	H17B—C17—H17C	109.5
C9—C8—H8B	111.2		
			
C16—N1—N2—C9	175.65 (9)	N1—N2—C9—C10	178.41 (8)
C7—N1—N2—C9	−6.89 (11)	N1—N2—C9—C8	−1.56 (11)
C6—C1—C2—C3	0.00 (16)	C7—C8—C9—N2	8.59 (11)
C1—C2—C3—C4	−0.79 (16)	C7—C8—C9—C10	−171.37 (9)
C1—C2—C3—Cl1	178.98 (8)	N2—C9—C10—C15	−178.72 (10)
C2—C3—C4—C5	1.16 (16)	C8—C9—C10—C15	1.24 (15)
Cl1—C3—C4—C5	−178.61 (8)	N2—C9—C10—C11	0.87 (14)
C3—C4—C5—C6	−0.76 (16)	C8—C9—C10—C11	−179.17 (10)
C4—C5—C6—C1	0.00 (15)	C15—C10—C11—C12	1.49 (15)
C4—C5—C6—C7	−178.94 (10)	C9—C10—C11—C12	−178.12 (9)
C2—C1—C6—C5	0.38 (15)	C10—C11—C12—C13	−0.15 (16)
C2—C1—C6—C7	179.32 (9)	C11—C12—C13—O1	177.34 (9)
C16—N1—C7—C6	67.68 (12)	C11—C12—C13—C14	−1.79 (15)
N2—N1—C7—C6	−109.72 (9)	O1—C13—C14—C15	−176.80 (10)
C16—N1—C7—C8	−171.01 (9)	C12—C13—C14—C15	2.36 (16)
N2—N1—C7—C8	11.59 (11)	C13—C14—C15—C10	−1.01 (16)
C5—C6—C7—N1	−120.77 (10)	C11—C10—C15—C14	−0.91 (15)
C1—C6—C7—N1	60.30 (12)	C9—C10—C15—C14	178.69 (10)
C5—C6—C7—C8	125.96 (10)	N2—N1—C16—O2	−178.73 (9)
C1—C6—C7—C8	−52.96 (13)	C7—N1—C16—O2	4.09 (15)
N1—C7—C8—C9	−11.08 (10)	N2—N1—C16—C17	1.74 (15)
C6—C7—C8—C9	108.36 (9)	C7—N1—C16—C17	−175.43 (9)

### Hydrogen-bond geometry (Å, º)

**Table:**

*D*—H···*A*	*D*—H	H···*A*	*D*···*A*	*D*—H···*A*
O1—H1*O*1···O2^i^	0.88 (2)	1.82 (2)	2.6971 (12)	171 (2)
C8—H8*A*···N2^ii^	0.99	2.56	3.5038 (14)	160
C8—H8*B*···O1^iii^	0.99	2.52	3.4887 (12)	166
C12—H12*A*···O2^i^	0.95	2.51	3.1982 (12)	130

Symmetry codes: (i) −*x*+3/2, −*y*+2, *z*+1/2; (ii) *x*−1, *y*, *z*; (iii) *x*−1/2, −*y*+3/2, −*z*+1.
